# Unveiling energy security in agriculture through vital indicators extraction and insights

**DOI:** 10.1038/s41598-024-59273-3

**Published:** 2024-04-15

**Authors:** Reihaneh Haghjoo, Shahla Choobchian, Enayat Abbasi

**Affiliations:** Department of Agricultural Extension and Education, Tarbiat Modares University, Tehran, 14115-111 Iran

**Keywords:** Economic growth, Environmental conservation, Energy consumption, Sustainability goals, Energy and society, Sustainability

## Abstract

Despite advancements in meeting various human needs, energy supply remains a top priority for all countries worldwide. The escalating energy consumption in the agricultural sector underscores the necessity to scrutinize its energy usage. Presently, there exists an absence of a precise tool for accurately measuring this consumption. Hence, this study aims to identify indicators for measuring energy security in agriculture, conducted in three phases: content analysis, indicator validation, and field investigation. In the content analysis phase, energy security indicators were extracted and grouped into four categories: accessibility, availability, utilization, and sustainability. Following this, a two-stage validation process led to the identification of 18 indicators for assessing energy security in agriculture. In the field phase, a tailored questionnaire was distributed to 160 randomly selected farmers. The findings revealed that the availability component held the highest rank in establishing energy security, with an average score of 3.31. However, the current status of the access component indicates a more unfavorable situation compared to other dimensions. Consequently, to achieve energy security in agriculture, particular emphasis should be placed on enhancing energy access. Key areas to address include reducing transportation costs and minimizing the use of chemical pesticides. This indicates a necessity for focused interventions aimed at improving both energy access and sustainability within the agricultural sector. These efforts would contribute to enhancing economic efficiency and promoting environmental conservation.

## Introduction

Energy is a critical factor in production, significantly contributing to the economic growth and development of nations. Its impact spans various sectors, from food production and transportation to the provision of medical and health services, as well as ensuring political stability. Consequently, energy security has emerged as a paramount concern, particularly in industrialized nations, over the past few decades. This heightened focus on energy security originated during the oil crisis in 1970, prompting numerous academic institutions to conduct studies analyzing the crisis's implications for the energy sector^1^. Since then, there has been increasing recognition of the importance of energy and its security in addressing various challenges^2^.

The International Energy Agency (IEA) defines energy security as "uninterrupted access to sufficient energy sources at reasonable and reliable prices"^3–6^. According to this definition, which is widely accepted among researchers, energy security encompasses three modes: long-term energy security, focusing on long-term investments to meet a country's energy needs in line with its economic development and sustainable environmental requirements, short-term energy security, emphasizing the ability of energy systems to rapidly respond to sudden changes in supply and demand; and lack of energy security, reflecting the economic and social impacts of energy, such as price fluctuations and lack of competition^7,8^.

The definition of energy security, coupled with the rapid increase in CO_2_ emissions from fossil fuels, highlights the significant environmental impact of energy consumption^9^. Moreover, agriculture contributes substantially to global warming through the production of greenhouse gases from activities such as tillage operations and methane emissions from livestock^10^. Additionally, the cost of producing crops is influenced by fuel prices^11^. Therefore, reducing reliance on fossil fuels and increasing the use of biofuels are key future objectives in the energy security sector. Numerous national and international studies have delved into energy security and related indicators. For example, in Taiwan, where fossil fuels dominate energy consumption, ensuring access to these resources for future generations is imperative^2^. Other researchers have stressed that diversifying energy resource usage alone is not enough to assess energy security, underscoring its broader impact on availability, affordability, and acceptability^12^. In a separate study, energy security was assessed across five dimensions: driving force, pressure, conditions, effects, and response, revealing significant pressure from economic growth, urbanization, and other factors, with adverse consequences for human life and the environment^13^.

In their examination of energy security indicators, Chiang Li et al. concluded that it can be evaluated across four dimensions: availability, accessibility, developability, and acceptance^14^. Another study evaluated energy security in West Africa using a specific component (investment) and five cross-sectoral components (governance, sustainability, reliability, affordability, and regional energy resources). Similarly, Yousefi et al. analyzed economic, social, political, and geopolitical indicators, concluding that comprehensive development necessitates leveraging local, national, regional, and international driving forces^15^.

Given agriculture's significant contribution to greenhouse gas emissions and environmental concerns associated with fossil fuel consumption, assessing energy security in this sector is crucial. However, specific indicators for this purpose have yet to be defined. Hence, the primary challenge of this study is identifying systematic indicators to operationalize various aspects of energy security towards sustainability. These indicators are vital for researchers and planners at governmental and private levels to effectively evaluate energy consumption methods.

Furthermore, energy security indicators serve as benchmarks for measuring progress toward sustainability and guide environmental programs and sustainable development goals. Without these indicators, accurately measuring and evaluating energy consumption practices is impossible. Therefore, the objective of this article is to identify and validate energy security indicators specific to the agricultural sector, providing valuable tools to assess energy security across different agricultural systems in Iran and globally.

This research progressed through three distinct stages, each contributing to a comprehensive understanding of the subject matter.

In the initial stage, a qualitative methodology was utilized to establish a foundational understanding. This involved exploring the nuances and complexities of the topic through methods such as content and thematic analysis.

Moving on to the second stage, a mixed-method approach was adopted, integrating both quantitative and qualitative methodologies. This allowed for a deeper exploration of the research questions by triangulating data from multiple sources, such as surveys and interviews.

Finally, in the third stage, the focus shifted exclusively to quantitative methodology. Here, statistical analysis was employed to identify patterns and relationships within the data. The quantitative aspect of the research was characterized as "non-experimental," indicating the absence of controlled variables, and "descriptive-correlation," highlighting the statistical operations used.

To collect data for this quantitative phase, a survey method was employed. Surveys were distributed to gather pertinent information on key variables of interest, enriching the overall findings of the research.

## Materials and methods

This article was conducted in three phases.

### First phase

This phase employed a descriptive-analytical approach, utilizing the inductive qualitative content analysis method outlined by Gal et al.^16^ for data analysis. Content analysis is a method enabling the systematic identification of properties within large amounts of textual information. This stage involved library studies and the extraction of energy security measurement indicators specific to the agricultural sector. To extract energy security indicators, 65 articles were reviewed. Table 1 lists the journals with the highest number of related articles. These articles were searched using keywords such as "energy security," "energy security index," and "renewable energy." To analyze the article content, main concepts related to energy security measurement were first extracted, termed open codes. These codes encompassed concepts specifically utilized in the examined texts. Following open coding, axial coding commenced. In axial coding, each basic code was compared with classes containing the measurement dimensions of these components and categorized accordingly. In the final step, after classification, the extracted dimensions and indicators were tailored to the agricultural sector, removing indicators not specific to this domain.

**Table 1:** Sources studied in the energy security sector.

Journal title	Number
*Energy*	10
*Environmental Management*	10
*Review of Renewable and Sustainable Energy*	8
*Asia Energy Research Center*	7
*Ecological Economics*	7
*Agricultural Economy and Development*	6
*Human Geography Researches*	5

### Second phase

This phase involves several key steps, including an initial survey of expert professors, preliminary modification and evaluation of the indicators, submission of the indicators to subject matter experts for assessing face and content validity (using CVI and CVR), and finalizing the indicators. A questionnaire was developed to assess energy security in the agricultural sector, incorporating insights from subject matter experts and indicators identified during the first phase. Initially, the questionnaire included 33 indicators. Subsequently, it underwent an initial evaluation by 10 subject matter experts using The Lincoln and Guba^17^ evaluation method to solicit feedback, which led to the deletion, modification, or alteration of certain items. Next, the revised indicators were presented to 30 subject matter experts in the form of a questionnaire containing 28 items. Ultimately, 22 experts expressed interest in completing the questionnaire, contributing to the refinement of the indicators.

#### Content validity of the questionnaire

Considering the amalgamation of definitions from various texts, content validity can be defined as "the extent to which the questions posed reflect the characteristics of the construct to be measured." The content validity index is the most commonly used quantitative method to ascertain content validity in multiple-choice scales. This method relies on the judgment of a panel of experts to assess the relevance of the items^18–20^.

In this study, the Lawshe method was employed to determine the content validity of the questionnaires quantitatively. This method involves calculating two coefficients: the content validity ratio (CVR) and the content validity index (CVI)^18^. To compute these coefficients, subject matter experts evaluate each item in terms of its necessity and relevance.

Content validity ratio (CVR): to calculate this coefficient, the indicators are presented to experts in the form of a questionnaire, and they are asked to rate the appropriateness of each indicator using a three-part spectrum: "necessary", "useful but unnecessary", and "unnecessary". Based on the experts' opinions, the content validity ratio for each indicator is determined using the following formula:

$$CVR \, = \, \frac{n - N/2}{{N/2 }}$$

In this formula: N is the total number of experts. n is the number of experts who have chosen the “necessary” option.

In the Lawshe method, the minimum acceptable CVR is determined based on the number of panel members. According to Table 2, the minimum CVR required for 22 panel members in this study is equal to 0.40.

**Table 2:** Coefficient of content validity ratio.

Number of experts	Value CVR	Number of experts	Value CVR	Number of experts	Value CVR
5	0.99	11	0.52	25	0.37
6	0.99	12	0.56	30	0.33
7	0.99	13	0.54	35	0.31
8	0.99	14	0.51	40	0.29
9	0.75	15	0.49	–	–
10	0.62	20	0.42	–	–

Ref.^42^.

#### Content validity index (CVI)

To calculate the CVI, experts are requested to provide their opinion on the indicators represented as items using a 4-point Likert scale: "completely relevant, needs little variation, needs a lot of variation, and irrelevant". The Content Validity Index (CVI) score for each statement is determined by dividing the number of experts who agree with ranks 3 and 4 (completely relevant and needs little change) by the total number of experts^20^. In the “mean” section of the content validity index, items with a CVI higher than 0.79 are confirmed^20,21^.

$${\text{CVI}}= \frac{\mathrm{total\, number\, of\, experts\, gave\, a\, score\, of\, }4\mathrm{ \, and\, }3\mathrm{ \, items}}{ \mathrm{total \, number\, of \, experts}}$$

### 3rd phase

In this stage of the research, the validated questionnaire from the previous phase was administered to the statistical samples. The questionnaire utilized a five-level Likert scale (ranging from very low = 1 to very high = 5) to assess energy security. The population under study in this phase comprised all "sampled farmers" (sample farmers are the individuals chosen based on specific criteria determined by the Iranian Agricultural Jihad Organization) in the agriculture and horticulture sectors between 2015 and 2019. The statistical population size was 270 "sampled farmers", with 160 individuals selected as the sample size according to Morgan's table.

For sampling in this phase of the research, the stratified random sampling method with proportional assignment was employed. This method is utilized when the target population of the research exhibits a heterogeneous and incongruous structure. Therefore, owing to the inconsistency and heterogeneity in the agricultural fields across the country, the research population was divided into different "strata." SPSS software was employed for data analysis. In this study, after data collection and classification, both descriptive and inferential statistical methods were utilized for data analysis. Descriptive statistics were employed to analyze the data for better characterization of demographic variables, including means, standard deviations, and variances. Additionally, a binomial test was employed to compare the current situation with the desired state, given the non-normality of the data.

In this study, several mitigation strategies were implemented to minimize bias, thereby ensuring the robustness and validity of the research findings. These strategies included:

1. Randomization: participants were randomly assigned to groups to reduce the risk of selection bias, ensuring that each group represented the larger population effectively.
2. Blinding: employing single-blind or double-blind designs helped prevent observer bias, as neither the participants nor the researchers were aware of certain critical aspects of the study, thus reducing the potential for biased assessments.
3. Standardized protocols: consistent procedures were meticulously followed during data collection to minimize variability and ensure reliability across all measurements and observations.
4. Pre-registration: hypotheses and analysis plans were specified in advance, before data collection commenced, thereby preventing post hoc decisions that could introduce bias based on the observed results.
5. Sensitivity analysis: various assumptions underlying the analysis were rigorously tested to evaluate their impact on the results, ensuring that the conclusions drawn were robust and not overly sensitive to specific conditions.
6. Transparent reporting: methods employed and potential biases were clearly documented, providing transparency and allowing readers to assess the reliability of the study's findings.
7. Peer review: the study design underwent critical evaluation by colleagues through peer review, providing valuable feedback and ensuring that potential biases were adequately addressed before publication.
8. External validation: findings were validated using independent datasets or external sources, confirming the consistency and generalizability of the results beyond the original study context.
9. Subgroup analysis: results were analyzed across different subgroups to identify any potential biases that may have influenced certain demographic or characteristic groups differently, thereby ensuring a comprehensive understanding of the findings.

By implementing these rigorous mitigation strategies, all potential biases were identified and appropriately addressed, thereby strengthening the credibility and reliability of the study's conclusions.

### Ethical approval

It should be mentioned that Authors identify: (a) Tarbiat Modares University’s ethical committee approved the research and the research have been performed in accordance with the Declaration of Helsinki. (b) Authors confirm that all research was performed by relevant guidelines/regulations. (c) All subjects gave informed consent for inclusion before participating in the study.

## Findings and discussion

### 1st phase: content analysis

**1st step of content analysis: open coding** : In this section, a comprehensive review was conducted on 65 scholarly articles about energy security, selected through systematic keyword searches utilizing terms such as "energy security", "energy security indicators", and "renewable energy". The essential concepts and content were meticulously extracted from these articles and subsequently organized into categories based on their thematic relevance and conceptual frameworks. Each discerned concept was methodically designated as a code, as delineated in Table 3.

**Table 3:** Open coding of energy security.

Categories	Indicators	Frequency
Availability	technologies for finding and extracting fossil fuels, exploitation of renewable resources, energy structure, primary energy supply index, resource estimation index; The availability of energy resources, the average ratio of reserves to production, self-sufficiency, and the share of national renewable energy supply, domestic energy reserves, and strategic energy reserves	22
Accessibility	Use of renewable energy, energy consumption from external sources, percentage of population with access to electricity, and percentage of population with access to new fuels for cooking and heating	14
Acceptable	Equitable access to quality energy resources Social and environmental concerns Share of CO_2_ emissions in global emissions	16
Energy affordability (financial power)	Non-carbon compact fuel basket (NCFP), oil cost per GDP, marginal cost of energy; ratio of price to gross national product, and prices of energy resources	25
Economic	Energy use, energy efficiency, supply efficiency, final cost of energy production, energy consumption, rational price of energy resources, profitability of activities, import dependence (import share in the energy basket), political stability (human resource development index), oil price (oil price), medium portfolio (electricity generating technology portfolio), carbon-free share (carbon-free fuels portfolio), market quality (index of access to energy carriers in the market), gross domestic product and… )	13
Stability	environmental protection, resource sustainability, resource security, fuel diversity, infrastructure adequacy, fuel diversity, preparedness for supply disruption, environmental sustainability, environmental sustainability; carbon-free share (basket of carbon-free fuels)	8
Policy	Policy frameworks, laws, government, trade, competition, dependence on foreign resources, political and economic stability of producers	11
Environmental	Emission of carbon dioxide gases, share of non-fossil fuel, share of electricity from total energy consumption	5
Technology development and productivity	Energy technologies, innovation and research Applying advanced technologies in the use of energy resources	6
Energy demand	Demand management, energy intensity (energy consumption per GDP)	2
Energy transport	International energy transportation, energy transportation cost, energy supply infrastructure	5
Resilience	Land use, climate change, pollution	3
Diversity index	Share of dominant fuel type in gross energy consumption	1
Social	Social effects, energy awareness	2

In the table above, the outcomes of open coding conducted on the contents of energy security articles reveal that energy security is assessed across 11 dimensions. Notably, "affordability" and "availability" emerge as pivotal dimensions, underscored by their recurrent mention in scholarly investigations. This underscores the substantial influence of affordability and availability on the broader discourse surrounding energy security. Additionally, other dimensions such as accessibility, utilization, economic and environmental considerations, technological advancements and efficiency, energy demand, energy distribution, and societal factors are identified.

**Second step: Axial coding: **Axial coding constitutes the subsequent phase of content analysis, aiming to establish interrelations among categories delineated during open coding. This stage entails juxtaposing each open code with classes encompassing the measurement dimensions of said indicators. The methodology involves organizing concepts and contents of indicators within each dimension, discerning overlaps and congruencies in meaning. Consequently, these indicators are classified into four primary categories of security dimensions, prioritized based on their frequency during the open coding phase. These core codes, namely availability, accessibility, utilization, and sustainability, are elucidated in Table 4.

**Table 4:** Core (Axial) coding of energy security.

Dimensions	Indicator	Frequency
Accessibility	The price of energy resources and their affordability The amount of fertilizer and pesticides used per hectare of the crop Supply and production efficiency using renewable energy (wind, solar, geothermal, water, biomass) and fossil fuels The cost of energy carriers Amount of energy consumption per hectare of crop The amount of use of advanced technologies in the use of renewable energy sources for product production	33
Availability	Availability of renewable energy The amount of self-sufficiency in renewable energy production The amount of renewable energy available to produce the product	29
Usability	Being acceptable in terms of quality, energy, and environmental pollution The amount of CO_2_ emissions in the renewable energy sector and environmental protection The amount of greenhouse gas emissions	17
Sustainability	Diversity in the use of renewable fuels State of Infrastructure adequacy Preparation for disruption in the supply of environmental sustainability (carbon-free fuel basket)	9

As illustrated in the table above, the measurement indicators of energy security have been delineated into four dimensions: accessibility, availability, utilization, and sustainability. Upon comparison, access to energy emerges as the predominant dimension, exhibiting a frequency of 33, followed by availability at 29, utilization at 17, and sustainability at 9. Subsequently, the primary dimensions of energy security are revealed individually.

#### Accessibility

The utilization of advanced technologies in energy consumption serves to facilitate access to energy resources^22^. This dimension, highlighted as the primary focus of this study, emphasizes the crucial role of infrastructure in ensuring sustainable and integrated energy provision. Key infrastructure elements encompass energy and electricity conversion facilities, particularly for water pumping within the agricultural sector^22^. Thus, the presence of suitable infrastructures, including renewable energy equipment such as solar and wind, stands as a prerequisite for delivering sustainable energy. Notably, these infrastructural investments emerge as pivotal components for ensuring energy accessibility in the agricultural domain^2,23–27^.

#### Availability

Diversification in energy resource utilization stands out as a key factor in ensuring the availability of energy resources^28,29^. This diversification encompasses the adoption of various energy resources and technologies, including both fossil fuels and clean energy resources, concurrently. Additionally, the integration of renewable energy resources and solar and wind equipment emerges as significant indicators of energy availability, particularly within the agricultural sector^2,22,23,27^.

#### Usability

Recognizing energy as a fundamental necessity of life, certain studies encompass social welfare within the ambit of energy security definitions. Usability, in this context, entails ensuring that energy utilized within the agricultural sector adheres to environmentally friendly practices, thereby minimizing pollution in water, soil, and food resources^2,23,24,27,30^.

#### Sustainability

The intertwined nature of sustainability and environmental concerns with energy stems from issues such as carbon emissions and the production of greenhouse gases, contributing to global warming and air pollution. The European Commission underscores the significance of environmental considerations and sustainability within energy security, emphasizing the importance of providing accessible, affordable, reliable, efficient, environmentally safe, and socially acceptable energy services^31^. The nexus between energy and the environment underscores the importance of transitioning away from fossil fuels, which pose threats to the environment, towards cleaner energy resources. Consequently, environmental protection has emerged as a focal point in global discourse. Notably, social and environmental considerations stand as critical components in shaping energy security paradigms in the contemporary era. Within the agricultural sector, the utilization of energy resources derived from animal and plant origins, such as biodiesel and biomass, holds promise for minimizing environmental pollution and atmospheric harm^22^.

### 3rd step: selective coding

This step of content analysis is the main step of coding and includes the process of integration and improvement of categories. At this stage, the extracted indicators were selected according to their nature and relevance in the agricultural sector, and the indicators that required revision were modified and improved. Also, the indicators that were irrelevant in the agricultural sector were deleted. Finally, in this stage of content analysis, where the final classification of categories was performed, energy security can be measured with 10 indicators in the form of four components (Table 5).

**Table 5:** The results of the third stage of content analysis.

Category	Indicators
Accessibility	The price of renewable energy sources and their affordability Cost of energy transportation The amount of use of oils, biofuels, chemical fertilizers, renewable and pesticides per hectare of the crop Amount of energy consumption per hectare of crop The amount of use of advanced technologies in the use of renewable energy sources for product production
Availability	Availability of renewable and non-renewable energy for product production Farmer's self-sufficiency level in renewable energy production
Usability	The state of quality of energy resources used in agriculture, gardens, and greenhouses (in terms of renewables) Amount of greenhouse gas emissions
Sustainability	Diversity in using renewable and non-renewable energy sources in the farm, for example: the use of organic fertilizers, biofuels, biofuels and carbon-free

The findings from the initial phase revealed that the energy security index is evaluated through four primary categories: accessibility, availability, utilization, and sustainability, each comprising its own set of indicators. Subsequently, validation was conducted in the second phase to assess the validity of the extracted indicators in evaluating the status of energy security within the agricultural sector.

### 2nd phase: validation

In this section, articles about the indicators of the four dimensions of energy security were scrutinized. Relevant items were extracted in correlation with these indicators. These items underwent thorough review and adjustments by subject matter experts. Subsequently, a set of 25 items across the four dimensions of energy security assessment was presented to a panel of experts comprising 30 university faculty members specializing in energy resource management. Ultimately, 22 experts consented to complete the questionnaire.

Upon receiving the completed questionnaires from the experts, the values of the content validity index and the coefficient of the content validity ratio were calculated for each index, alongside their respective averages. Some indicators that necessitated modifications based on expert feedback were revised, while others were removed. Ultimately, the total average of the content validity ratio coefficient, as per the Lawshe table, exceeded 0.42, indicating an acceptable value. Moreover, the content validity index of the questionnaire surpassed 0.8, further affirming its acceptability.

The outcomes presented in the Table 6 demonstrate that all remaining indicators meet the minimum required criteria for Content Validity Ratio (CVR) and Content Validity Index (CVI), affirming their adequacy in measuring energy indicators within the agricultural sector. The study findings reveal that energy security is evaluated across four dimensions: accessibility, availability, utilization, and sustainability.

**Table 6:** Validation of energy security indicators.

Category	Indicators	CVR	CVI
Accessibility	Energy spent to transport the product to the consumer market Energy spent on transporting farm inputs Use of biofuels (animal waste, agricultural and forest waste, etc.) Use of chemical fertilizers (nitrogen, phosphate, nitrogen) Use of human power (labor force) Use of agricultural machines and tools Use of chemical poisons (pesticides, herbicides, fungicides, etc.)	0.43 0.50 0.50 0.64 0.43 0.45 0.50 0.40	0.86 0.80 0.81 0.79 0.79 0.82 0.79 0.86
Availability	Using wind energy sources (turbine, water pumping) Use of solar energy sources (solar dryer, photovoltaic systems) Use of geothermal energy sources (hot underground water for heating) Use of fossil fuels (tractor, tiller, combine, etc.) Use of electricity for the power drive of irrigation electro-pumps Use of renewable energy equipment (solar, wind and water)	0.50 0.68 0.64 0.43 0.50	0.82 0.92 0.86 0.79 0.82
Usability	Application of necessary training in line with energy security (advantages of using clean energies, etc.) The ability to get government support in investments using clean (renewable) energies Using loans and facilities for renewable energy resources equipment	0.43 0.45 0.45	0.80 0.79 0.86
Sustainability	Bio-energy production (animal waste, agricultural and forest waste, etc Use of diesel fuel	0.68 0.43	0.80 0.79

Within the accessibility dimension, the indicator "energy spent to produce and transport the product" exhibits high credibility, consistent with the findings of Khodadoostan^32^. Concerning the availability index "the use of clean energy, especially solar energy for extracting irrigation water," demonstrates a high content validity ratio and content validity index. As a result, the indicators are instrumental in mitigating the consumption of non-renewable energy, consistent with the research of Zahedi et al.^33^ and Khodadostan^32^. Consequently, there is a need for incentives to incentivize farmers to adopt and produce renewable energy.

Furthermore, the index "Government support in clean (renewable) energy investments" attains an acceptable and high Content Validity Ratio and Content Validity Index, echoing the findings of Eidi^34^. Accordingly, policy measures should be implemented to elevate the cost of fossil fuel usage.

In terms of sustainability, "Using necessary training in the direction of energy security (benefits of using clean energy, etc.)" emerges as a relatively valid index for fostering farmers' energy resource security within the agricultural sector, aligning with the findings of Bojnec and Papler^35^, Sezgin et al.^36^, and Lolavar and Niknami^37^. Addressing the lack of specialized human resources through training initiatives is imperative.

Moreover, indices such as "use of fossil fuel (availability dimension), the cost spent on transportation and the use of fertilizers and pesticides (availability dimension)" exhibit high face and Content Validity Index coefficients, consistent with the findings of Mohammadi Ilar et al.^38^, Ehsani and Shahrbannejad^39^, and Taghinejad^40^. Incentivizing renewable energy production stands out as a viable solution, aimed at bridging the gap between conventional fuel costs and renewable energy.

The index of "use of geothermal energy resources (availability dimension)" is in line with the findings of Heidari^41^. Additionally, the study underscores the significant impact of chemical fertilizers and fuel components on energy consumption, with chemical fertilizers exhibiting the highest consumption rate and labor energy the lowest. Consequently, prioritizing these indicators in energy resource security assessments by farmers holds paramount importance for crop production, offering a potential avenue to enhance energy resource security within the agricultural sector.

### 3rd phase

#### Ranking and analysis of the gap between the present and ideal status of Accessibility component items

In the third phase, the results of the ranking and analysis of the gap between the existing and ideal situations of items related to the access component are presented in Table 7. The findings reveal that, in the current scenario, the utilization of human power (labor force) ranks first with an average score of 3.83, followed by the use of machinery and agricultural tools with an average score of 3.77, and the minimal energy expended in transporting products to the consumer market, ranking third with an average score of 2.86. Conversely, the utilization of biofuels (animal waste, agricultural and forest waste, etc.) ranks last with an average score of 1.62.

**Table 7:** Status of managerial evaluation of energy security element of sample farmers.

Item	Code	Components	Descriptive statistics			Inferential statistics (binomial test)				
			Mean	Standard deviation	Rank	Ideal limit	N	Observed proportion	Hypothesized Proportion	Significance level
Accessibility	ES.Av4	Use of human power (labor force)	3.38	0.901	1	≤ 4	125	0.78	0.50	0.001
						> 4	35	0.22		
	ES.Av5	Use of agricultural machines and tools	3.77	1.017	2	≤ 4	123	0.77	0.50	0.001
						> 4	37	0.23		
	ES.Av6	Energy spent to transport the product to the consumer market*	2.86	1.069	3	≤ 4	145	0.91	0.50	0.001
						> 4	15	0.09		
	ES.Av2	Use of chemical poisons (pesticides, herbicides, fungicides…)*	2.79	1.122	4	≤ 4	141	0.88	0.50	0.001
						> 4	19	0.12		
	ES.Av7	Energy spent for transporting farm inputs*	2.73	0.958	5	≤ 4	154	0.96	0.50	0.001
						> 4	6	0.04		
	ES.Av3	Use of chemical fertilizers (nitrogen, phosphate, and potash)*	2.46	0.954	6	≤ 4	153	0.96	0.50	0.001
						> 4	7	0.04		
	ES.Av1	Use of biofuels (animal waste, agricultural and forest waste, etc.)	1.62	1.027	7	≤ 4	154	0.96	0.50	0.001
						> 4	6	0.04		
Availability	ES.Ac13	Use of electricity for driving power of irrigation electro pumps (water pumping)*	3.51	1.449	1	≤ 4	109	0.68	0.50	0.001
						> 4	51	0.32		
	ES.Ac11	Use of fossil fuels (tractor, tiller, combine, etc.)*	2.59	1.472	2	≤ 4	130	0.81	0.50	0.001
						> 4	30	0.19		
	ES.Ac12	Use of renewable energy equipment (solar, wind and hydro)	1.62	0.924	3	≤ 4	156	0.97	0.50	0.001
						> 4	4	0.03		
	ES.Ac8	Use of wind energy resources (turbine, water pumping)	1.53	1.121	4	≤ 4	153	0.96	0.50	0.001
						> 4	7	0.04		
	ES.Ac9	Use of solar energy sources (solar dryer and photovoltaic systems)	1.21	0.615	5	≤ 4	159	0.99	0.50	0.001
						> 4	1	0.01		
	ES.Ac10	Use of geothermal energy sources (underground hot water in heating)	1.19	0.495	6	≤ 4	160	0.99	0.50	0.001
						> 4	0	0.01		
Usability	ES.U14	Applying the necessary training in the direction of energy security (advantages of using clean energy, etc.)	2.77	1.309	1	≤ 4	138	0.86	0.50	0.001
						> 4	22	0.14		
	ES.U15	The ability to get government support for clean (renewable) energy investments	2.16	1.154	2	≤ 4	151	0.94	0.50	0.001
						> 4	9	0.06		
	ES.U16	Use of loans and facilities for equipment of renewable energy resources	2.09	1.151	3	≤ 4	155	0.97	0.50	0.001
						> 4	5	0.03		
Sustainability	ES.S18	Using diesel fuels*	2.87	1.424	1	≤ 4	130	0.81	0.50	0.001
						> 4	30	0.19		
	ES.S17	Bioenergy production (livestock waste, agricultural and forest waste, etc.)	2.21	1.343	2	≤ 4	146	0.91	0.50	0.001
						> 4	14	0.19		
* Reverse										

In the analysis of the gap between the existing and ideal situations, the results indicate a significant disparity across all items within the access component. Specifically, the score of the ideal situation (assumed numbers greater than 4) markedly exceeds the score of the existing situation for all items pertaining to the access component. Notably, the widest gap between the ideal and existing situations is observed in the utilization of biofuels (animal waste, agricultural and forest waste, etc.), as evidenced by the average score of 1.62 and the presence of a substantial difference.

#### Ranking and analyzing the gap between the existing and the ideal of the items of the Availability component

The ranking and analysis of the disparity between the existing situation and the ideal state of items related to the availability component are presented in Table 7. According to the findings, in the current scenario, the use of electricity to power electric irrigation pumps (for water pumping) ranks first with an average score of 3.51, followed by the utilization of fossil fuels (for machinery like tractors, tillers, combines, etc.) ranking second with an average score of 2.59. Conversely, the utilization of geothermal energy sources (specifically underground hot water for heating) ranks last with an average score of 1.19.

In the analysis of the gap between the existing and ideal situations, the results indicate a substantial disparity across all items within the availability component. Notably, the score of the ideal situation (assumed to be greater than 4) significantly exceeds the score of the existing situation for all items related to the availability component. The widest gap between the ideal and existing situations is observed in the utilization of geothermal energy sources (underground hot water for heating), as evidenced by the average score of 1.19 and the presence of a significant difference. In other words, the discrepancy between the ideal situation and the current state of utilizing geothermal energy resources (underground hot water for heating) is the most pronounced compared to the gap between the ideal state and the current state of other items.

#### Ranking and analysis of the gap between the existing and the ideal of usability component items

The ranking and analysis of the discrepancy between the existing situation and the ideal state of items related to the usability component are illustrated in Table 7. According to the findings, in the current context, the utilization of necessary training focused on energy security (including the benefits of using clean energy, etc.) ranks first with an average score of 2.77, while the utilization of loans and facilities for renewable energy equipment ranks last with an average score of 0.92.

In examining the gap between the existing and ideal situations, significant disparities are observed across all usability component items. Specifically, the score of the ideal situation (assumed to be greater than 4) substantially exceeds the score of the existing situation for all items within the usability component. Notably, the most notable gap is observed in the utilization of loans and facilities for renewable energy equipment, as indicated by the average score of 2.09 and the presence of a significant difference. In essence, the discrepancy between the ideal and current status of utilizing loans and facilities for renewable energy equipment is the most prominent compared to the gap between the ideal and current status of other items.

#### Ranking and analysis of the gap between the current and ideal status of sustainability component items

The ranking and analysis of the disparity between the existing situation and the ideal state of items related to the sustainability component are presented in Table 7. The results indicate that, in the current context, the lower utilization of diesel fuels, with an average score of 2.87, and the production of biological energy (such as animal waste, agricultural and forest waste, etc.), with an average score of 2.21, are indicative of an unfavorable situation.

Upon examining the gap between the existing and ideal situations, significant differences are noted between the sustainability component items and their ideal counterparts. Specifically, these items exhibit scores significantly lower than the assumed ideal threshold of 4, indicating a notable disparity from the desired state.

#### Ranking and analyzing the gap between the current and ideal status of energy security components

Table 8 presents the ranking and analysis of the gap between the current and ideal statuses of energy security components. The findings reveal that, in the current scenario, the availability component holds the highest rank with an average score of 3.31, followed by the sustainability component with an average score of 2.74.

**Table 8:** State of managerial assessment of energy security of sample farmers.

Code	Components	Descriptive statistics			Inferential statistics (binomial test)				
		Mean	Standard deviation	Rank	Ideal limit	N	Observed proportion	Hypothesized proportion	Significance
ES.Av	Availability	3.31	0.540	1	≤ 4	156	0.98	0.50	0.001
					> 4	4	0.02		
ES.S	Stability	2.74	1.054	2	≤ 4	151	0.96	0.50	0.001
					> 4	9	0.06		
ES.U	Usability	2.66	1.034	3	≤ 4	154	0.96	0.50	0.001
					> 4	6	0.04		
ES.Ac	Access	2.34	0.559	4	≤ 4	160	100	0.50	0.001
					> 4	0	0		

Upon analyzing the disparity between the existing and ideal situations, it becomes evident that a significant difference exists across all components of energy security. Each component's current status falls considerably below the ideal level. In essence, the score of the ideal situation (assumed to be greater than 4) significantly surpasses the score of the current situation for all components of the energy security framework.

Notably, the access component exhibits the most substantial gap between the ideal and existing statuses, as evidenced by the average score of 2.34 and the presence of a significant difference. This discrepancy in the access component's status represents the widest gap compared to the other components.

**Figure 1:**
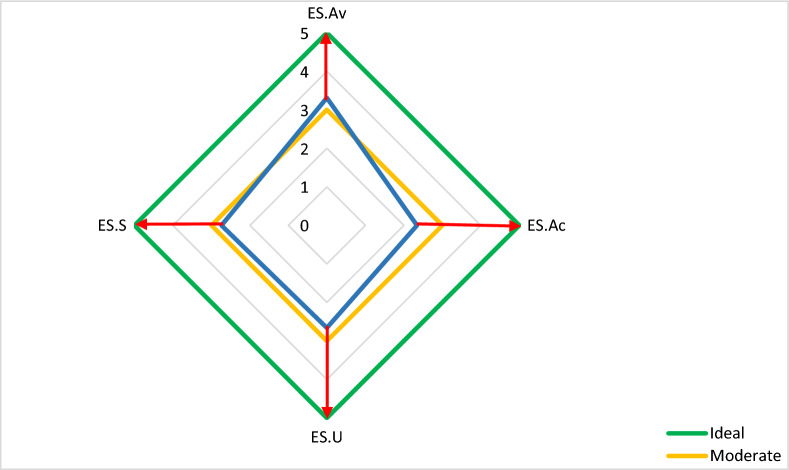
Status of managerial assessment of energy security of sample farmers.

The disparity between the components of energy security in the existing and ideal situations is illustrated in Fig. 1. As depicted, among the four indicators of energy security, the largest gap exists between the current and desired conditions of the components of accessibility and sustainability, while the smallest gap pertains to the availability component. These results suggest that farmers have largely aligned their practices with energy sustainability principles in the production process.

## Conclusion 

The purpose of this article stems from the extensive research conducted globally on energy security. Thus, our approach involved employing content analysis of relevant texts and articles to extract various dimensions of energy security. Subsequently, these dimensions and indicators underwent validation by subject experts using the Lawshe method, a common approach for assessing research validity. Finally, farmers evaluated these indicators via a validated questionnaire, and the results were analyzed using SPSS software.

Among the four indicators of energy security, the availability and sustainability components exhibit the smallest gap from the desired state. Analysis of the availability component revealed that a majority of “sampled farmers” predominantly utilize electricity as the driving force for irrigation electro pumps, a renewable energy source. This underscores the commendable performance of farmers in this realm.

The stability component also demonstrates satisfactory performance. Examination of its items indicates that leading farmers judiciously utilize diesel fuels, derived from fossil fuels. Given the environmental and sustainability concerns associated with fossil fuels, it is imperative to minimize their usage.

Regarding the usability component, analysis suggests that while “sampled farmers” have relatively implemented the training they received towards adopting clean energy practices, their utilization of loans and facilities for renewable energy equipment remains suboptimal. This implies that while farmers possess the necessary information about clean energy, they lack the requisite resources and infrastructure for its effective utilization.

Finally, analysis of the access component, identified as the weakest aspect in establishing energy security, indicates that “sampled farmers” effectively employ manpower and agricultural tools in the production process. However, there is a notable underutilization of biofuels in favor of conventional fuels. Given the adverse environmental effects and finite nature of fossil fuels, it is crucial to raise awareness among farmers about the benefits of biofuel usage to mitigate this gap.

According to the research findings, several proposed solutions are suggested to farmers to establish energy security:

1. Enhancing energy resource efficiency by optimizing the consumption of various inputs in the system through proper selection of type, quantity, method, and timing of consumption. This includes increasing the efficiency of inputs such as fertilizers and pesticides to reduce energy consumption on the farm.
2. Utilizing green manure and implementing crop rotation, particularly alternating wheat crops with pulses, to minimize the need for nitrogen fertilizers.
3. Adopting combined machinery capable of multitasking (e.g., fertilizing and seeding) and implementing minimal and conservation tillage practices to reduce fossil fuel consumption by minimizing machinery usage and reducing soil disturbance. Maintaining crop residue on soil surfaces through minimum tillage systems also reduces the need for chemical fertilizers.
4. Advocating for government institutions to play a vital role in informing and supporting the adoption of new technologies. This involves implementing promotion and incentive policies to encourage farmers to transition from fossil fuels to renewable energy sources.
5. Providing special facilities and offering low-interest loans through defined financial resources to support renewable energy adoption in rural areas and the agricultural sector.
6. Conducting pilot projects to showcase the benefits and opportunities of new energy sources, which can effectively build trust and encourage farmers to embrace renewable energy solutions.
7. Facilitating communication between agricultural research departments and farmers with designers and implementers of solar energy technologies. Collaboration in implementing proposed measures can enhance energy security, improve farmer performance, and contribute to sustainability goals.

While the proposed solutions offer promising avenues for enhancing energy security in agriculture, it is imperative to acknowledge the inherent limitations of this research. Firstly, the dynamic nature of agricultural systems and the evolving landscape of energy technologies may introduce uncertainties regarding the long-term effectiveness of the proposed solutions. Additionally, the intricate interactions between different components of the agricultural ecosystem and energy infrastructure may pose challenges in accurately assessing the impact of interventions. To address these limitations and further advance our understanding of energy security in agriculture, future research could explore several innovative avenues. Firstly, employing advanced modeling techniques and scenario analysis to anticipate the potential outcomes of energy security interventions under varying environmental and socio-economic conditions would provide valuable insights. Additionally, leveraging emerging technologies such as remote sensing and precision agriculture could enable more precise monitoring and management of energy use in agricultural operations. Furthermore, interdisciplinary research approaches that integrate insights from fields such as ecology, economics, and sociology could offer a comprehensive understanding of the complex interactions shaping energy security in agriculture. Lastly, exploring innovative policy frameworks and institutional arrangements to foster collaboration between stakeholders and facilitate the adoption of sustainable energy practices in agriculture could pave the way for transformative solutions in the future.

Based on the recommendations provided, emerging trends in agricultural energy security research, which identify and explore novel aspects at the farm level, may encompass:

1. Adoption of digital technologies: integrate digital tools like Internet of Things (IoT), artificial intelligence (AI), and block-chain to efficiently monitor and manage energy consumption on farms. This may entail developing smart sensors and platforms to optimize resource usage and minimize wastage.
2. Exploration of bioenergy solutions: investigate innovative bioenergy alternatives such as anaerobic digestion of agricultural waste or cultivating energy-dedicated crops for on-farm energy generation. Research efforts could concentrate on enhancing the efficiency and scalability of bioenergy systems while ensuring minimal environmental impact.
3. Study of climate-resilient agriculture: examine energy-efficient farming practices that enhance resilience to climate change, like implementing agroforestry systems or precision irrigation technologies. These methods could mitigate the effects of climate variability on energy availability and agricultural productivity.
4. Promotion of circular economy principles: highlight the importance of circular economy principles to reduce energy inputs and maximize resource efficiency in agricultural operations. This may involve deploying closed-loop systems for nutrient management, water recycling, and extracting energy from agricultural by-products.
5. Encouragement of community-based energy initiatives: Foster community-led energy initiatives empowering farmers to invest collectively in renewable energy infrastructure and resource-sharing. This might entail establishing cooperatives or partnerships to facilitate the adoption of renewable energy technologies at a local level.
6. Utilization of behavioral insights and decision support systems: employ behavioral insights and decision support systems to motivate farmers towards adopting energy-efficient practices. Research could delve into understanding farmers' attitudes, motivations, and barriers to embracing sustainable energy technologies, crafting targeted interventions for behavior change.
7. Assessment of resilient supply chains: evaluate the resilience of energy supply chains in agriculture and explore decentralized energy solutions to mitigate disruptions. This could involve studying alternative energy sources, storage technologies, and distribution networks to ensure reliable energy access for farming operations.
8. Exploration of policy innovation and stakeholder engagement: investigate innovative policy frameworks and governance mechanisms to incentivize investments in energy security within agriculture. Research could analyze the role of government policies, financial incentives, and stakeholder engagement strategies in facilitating the transition to sustainable energy practices on farms.

By addressing these emerging trends and exploring new aspects of agricultural energy security research, stakeholders can progress towards enhancing energy resilience, improving resource efficiency, and promoting sustainability in agricultural production.

## Research limitations and future research directions

This research, akin to other studies, encounters certain limitations:

1. Sectoral exclusivity: this research was confined solely to the agricultural and horticultural sector, limiting its generalizability to other sub-sectors within the broader agricultural industry. As a result, the findings may not be directly applicable to areas such as greenhouse cultivation or livestock farming.
2. Sampling bias: the focus on a specific set of sample farmers during the measurement phase introduces potential bias when attempting to generalize the findings to the entire farming population. Caution is warranted in extrapolating the results to all farmers, as the sample may not fully represent the diversity within the farming community.
3. Indicator customization: the indicators identified in this study are tailored specifically for farmers in the agriculture and orchard sectors. Consequently, their transferability to different agricultural domains, such as greenhouse management or livestock farming, may be limited. This restricts the broader applicability of the identified indicators across diverse agricultural settings.

Future study suggestions are provided as follows:

1. Broader sectoral representation: future research endeavors should aim to encompass a wider range of agricultural sub-sectors beyond agriculture and horticulture. By including sectors like greenhouse cultivation, livestock farming, aquaculture, etc., studies can provide more comprehensive insights into energy security issues across the entire agricultural industry.
2. Diverse sampling strategies: implementing diverse sampling strategies that capture a more representative sample of the farming population can help mitigate sampling bias. This may involve employing stratified sampling techniques or ensuring adequate representation from various geographic regions and farm sizes.
3. Universal indicator development: prioritizing the development of indicators that are universally applicable across different agricultural settings is essential. Future studies should focus on extracting indicators that can effectively measure energy security in diverse contexts, facilitating comparisons and benchmarking across different agricultural domains.
4. Interdisciplinary collaboration: encouraging interdisciplinary collaboration between researchers from agronomy, engineering, economics, and other relevant fields can enrich energy security research in agriculture. This collaborative approach can foster the development of holistic measurement frameworks and solutions that address the unique challenges faced by various agricultural sectors.
5. Longitudinal studies: conducting longitudinal studies that track changes in energy security indicators over time can provide valuable insights into trends and dynamics within the agricultural industry. Long-term monitoring can help identify emerging challenges and inform policy and practice interventions effectively.

By addressing these suggestions, future research endeavors can overcome the limitations of this study and contribute to advancing knowledge and understanding of energy security issues in agriculture across diverse settings.

## Data Availability

The datasets used and/or analysed during the current study available from the corresponding author on reasonable request.
